# Investigations of Accessibility of T2/T3 Copper Center of Two-Domain Laccase from *Streptomyces griseoflavus* Ac-993

**DOI:** 10.3390/ijms20133184

**Published:** 2019-06-28

**Authors:** Azat Gabdulkhakov, Ilya Kolyadenko, Olga Kostareva, Alisa Mikhaylina, Paulo Oliveira, Paula Tamagnini, Alexander Lisov, Svetlana Tishchenko

**Affiliations:** 1 Institute of Protein Research RAS, Institutskaya 4, Pushchino, Moscow 142290, Russia; 2 i3S—Instituto de Investigação e Inovação em Saúde, Universidade do Porto, 4200-135 Porto, Portugal; 3 IBMC—Instituto de Biologia Molecular e Celular, Universidade do Porto, 4200-135 Porto, Portugal; 4 Departamento de Biologia, Faculdade de Ciências da Universidade do Porto, 4169-007 Porto, Portugal; 5 G.K. Skryabin Institute of Biochemistry and Physiology of Microorganisms, Russian Academy of Sciences, Pushchino 142292, Moscow Region, Russia

**Keywords:** two-domain laccases, crystal structures, T2/T3 copper site, tunnels

## Abstract

Laccases (EC 1.10.3.2) are multicopper oxidoreductases acting on diphenols and related substances. Laccases are highly important for biotechnology and environmental remediation. These enzymes contain mononuclear one T2 copper ion and two T3 copper ions (Cu3_α_ and Cu3_β_), which form the so-called trinuclear center (TNC). Along with the typical three-domain laccases Bacteria produce two-domain (2D) enzymes, which are active at neutral and basic pH, thermostable, and resistant to inhibitors. In this work we present the comparative analysis of crystal structures and catalytic properties of recombinant 2D laccase from *Streptomyces griseoflavus* Ac-993 (SgfSL) and its four mutant forms with replacements of two amino acid residues, located at the narrowing of the presumable T3-solvent tunnels. We obtained inactive enzymes with substitutions of His165, with Phe, and Ile170 with Ala or Phe. His165Ala variant was more active than the wild type. We suggest that His165 is a “gateway” at the O_2_-tunnel leading from solvent to the Cu3_β_ of the enzyme. The side chain of Ile170 could be indirectly involved in the coordination of copper ions at the T3 center by maintaining the position of the imidazole ring of His157 that belongs to the first coordination sphere of Cu3_α_.

## 1. Introduction

Laccases (benzenediol:oxygen oxidoreductases) are copper-containing enzymes that catalyze the oxidation of diverse compounds by molecular oxygen. They are involved in biological processes, such as lignification of plant cell walls and lignin degradation, heavy metal homeostasis and spore morphology. During oxidation by laccases, oxygen is reduced to water, through the transfer of four electrons. The enzyme is used for the transformation of xenobiotics, delignification, and the oxidation of substances of industrial interest (e.g., textile dyes), as well as in cosmetics and medicine [1].

The active center of laccases contains four copper ions: A blue (Cu1) mononuclear copper center (T1) and a trinuclear copper cluster (T2/T3); the latter consists of the mononuclear T2 copper ion (Cu2) and two T3 copper ions (Cu3_α_ and Cu3_β_) [2]. T1 acts as a center for the reception of electrons from the reducing substrate, while the TNC serves as a binding site for the molecular oxygen and its reduction to water [3].

Three-domain (3D) laccases are monomers consisting of three cupredoxin-type domains [4]. two-domain (2D) laccases consist of two domains and exist as homotrimer, with the T2/T3 located at the interface of domain 2 and domain 1 of neighboring molecules, and T1 located near the surface of domain 2 [5].

The TNC plays an important role in functioning of laccases. There are indications that the T1 center is not absolutely required for the activity [6]. The authors have shown that the deletion of domain 3, which contains the T1 copper center from a bacterial 3D laccase does not abolish the activity of the enzyme. The catalytic cycle of 3D and 2D laccases is initiated by the oxidation of a substrate near the T1 center, four electrons produced by the oxidation of four molecules of substrate are sequentially transferred from the Cu1 to the TNC, where an oxygen molecule is reduced to water to complete the cycle. The presumed role of Cu2 and Cu3 ions is to anchor the dioxygen molecule to the TNC and transient binding of the OH^−^ groups before the addition of a proton and release of water into the outlet tunnel [7].

As TNC is located inside the protein, laccases must contain tunnels providing access of molecular oxygen to the T2/T3 and removal of water. We determined three-dimensional structures of recombinant 2D laccases from *Streptomyces viridochromogenes* Ac-629 [8] and *S. griseoflavus* Ac-993 (SgfSL) [9]. Comparative structural analysis of TNC environments of 3D laccases and 2D laccases showed differences in substrate/product transport network, connecting T2/T3 center with surface of the protein [10]. In 3D laccases wide, clearly defined tunnels between the surface and the trinuclear site provide access to the center during the catalytic cycle. While, 2D laccases tunnel(s) are not clearly defined.

In this study, SgfSL has been used as a model system to determine the transport tunnels connecting the TNC with surface. Based on our results, we propose that mobile positively charged side chain of histidine 165 acts as a “gate” in the tunnel that transports oxygen molecules to T2/T3 center.

## 2. Results

### 2.1. Structural Comparison of Tunnels of Three-Domain and Two-Domain Laccases

The structural analysis indicates that the trinuclear cluster [11] and tunnels leading toward the TNC, in 2D and 3D laccases, are different. In structures of 2D laccases copper ions of the T3 center are coordinated by six Nε atoms of histidine residues and not by five Nε and one Nδ (like in 3D laccases). Furthermore, tunnel oriented toward the T2 center in 2DLac has a tyrosine residue—a donor of electrons [11]. In the 3D laccases, the structural position of the OH^−^ group of this residue is occupied by a water molecule. The 3D laccases have two well-defined tunnels approaching the TNC and freely accessible to solvent. As an example, we used the structure of 3D laccase from *Steccherinum murashkinskyi* with currently the highest resolution for 3D laccases – 0.95Å (PDB ID 5E9N). The tunnel leading towards the Cu2 (T2 tunnel) can be responsible for the transport of protons to the active center [12]. The role of the tunnel leading towards the T3 center (T3 tunnel) is probably the transport of molecular oxygen [13,14]. The minimum radius of T3 tunnel of 3DLac *S. murashkinskyi* is sufficient for passing of oxygen molecules (1.38 ± 0.01 Å). The T2 tunnel is rather narrower (the minimum radius is 1.13 ± 0.01 Å).

The physical properties of xenon and krypton make them particularly good analogs of dioxygen because of their solubility in hydrophobic environments, and their van der Waals radii are comparable to that of O_2_. Xenon and/or krypton were used similarly to identify hydrophobic cavities and possible tunnels for oxygen transport in different proteins [15,16,17,18].

To find the possible pathways for dioxygen transfer to TNC experimentally, we determined the structures of SgfSL wt derivatized with xenon or krypton. Crystals of SgfSL wt, complexed with xenon or krypton, were obtained using both techniques of Xenon Chamber (Hampton Research) and “soak-and-freeze” methodology at 150 bar pressure [19]. Unfortunately, xenon and krypton-binding sites were only observed within the hydrophobic core of the molecule, and none in the vicinity of TNC.

**Figure 1:**
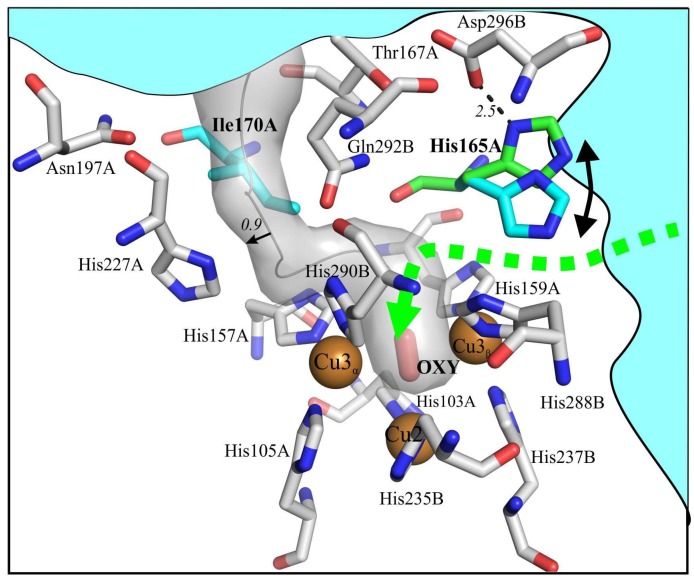
Estimated oxygen tunnels in the SgflSL WT (PDB entry 5LHL). The calculated trajectory of the T3_α_ tunnel behind Ile170 is shown in gray. The trajectory of the T3_β_ tunnel, for which His165 serves as the “gateway”, is green. The side chains of His165 is blue in the structure (“close” state) and green in possible open position. OXY – dioxigen. The distances are shown in angstroms. Bulk solvent is shown in cyan. Chains A and B belong to the neighboring monomer.

The structural analysis shows that the T2/T3 center in 2D laccases are less accessible. In these structures, the chains of water molecules are interrupted by side chains of amino acid residues, which apparently can play the role of a “gateway”. Using program *Caver* [20], we calculated one T3 tunnel (we referred to it as T3_α_) closely approaching the TNC. The side chain of Ile170 significantly narrowed this tunnel. The second tunnel (T3_β_) emerged upon rotation on the side chain of His165, which closed the shortest route from the protein surface to the cavity between Cu3_α_ and Cu3_β_ (Figure 1). Taking into account the thermal shaking calculations showed that these tunnels are potentially suitable for the passage of dioxygen.

The existence of a tunnel leading through the central cavity to this histidine was assumed earlier for the laccase of *S. coelicolor* [5]. In all the available structures of 2D laccases, the histidine is observed in a “closed” conformation and width of the proposed tunnel is 0.71 ± 0.09 Å. We performed a modeling of the sterically resolved state of the side chain of His165 with the hydrogen bonding between the histidine and Asp296 of the neighboring monomer. The resulting displacement widens the tunnel to 1.28 Å, which is sufficient for passing of dioxygen. We suggest that “open” and “closed” states of the proposed T3_β_ tunnel are provided by the mobility of the imidazole ring His165.

### 2.2. SgfSL and Mutants: Crystal Structures and Catalytic Activity

To assess the accessibility of TNC, we constructed and purified mutant variants of SgfSL by replacing the amino acids, His165 or Ile170, to either Phe or Ala.

**Figure 2:**
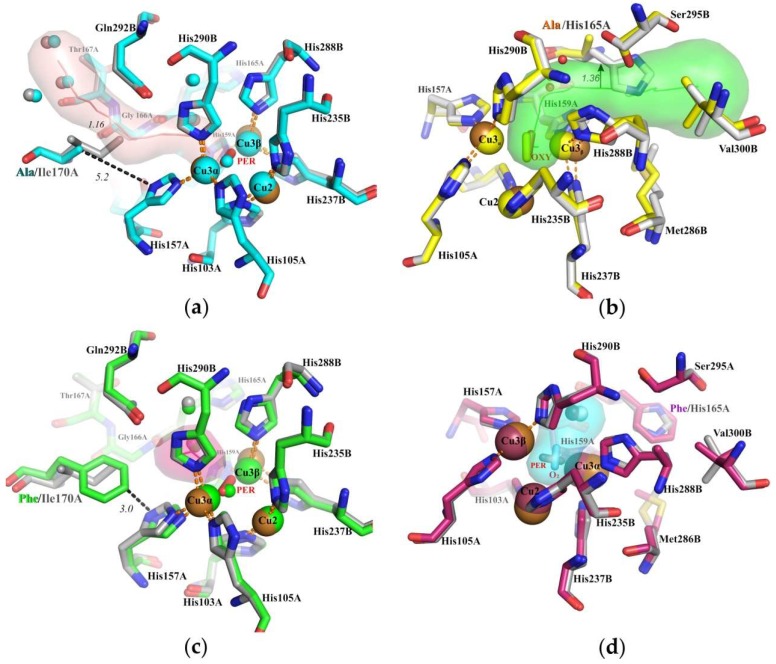
Structure of the T2/T3 center of SgfSL Ile170Ala variant (**a**), His165Ala (**b**), Ile170Phe (**c**) and His165Phe (**d**). PER - peroxide ion, OXY - dioxigen. Copper ions are shown as spheres. The distances between atoms are in angstroms. Residues of the wild type protein are shown in gray, residues of mutant proteins are shown in various colors. The trajectories of tunnels are shown as colored semi-transparent shapes.

We determined the crystal structures of the four mutants and compared them with the structure of the wild type protein. The most important structural parameters are summarized in Table 1, the ligands bound to the TNC, presented in Table S1, and the examples of electron density maps shown in Figure S1. The structures of the mutants confirm the desired single amino acid replacements; no significant changes in the overall fold of the alpha-carbon chain of the enzyme or in the vicinity of the active sites were observed. The r.m.s.d. parameters have never exceeded 0.4 Å. Significant changes occurred only in the areas of point mutations. In the case of substitutions for phenylalanine (H165F and I170F), the tunnels were closed (Figure 2c,d). When replacing isoleucine by alanine at position 170, the T3_α_ tunnel was expanded to 1.16 ± 0.11 Å (Figure 2a), the H165A mutation expanded the T3_β_ tunnel even more, to 1.36 ± 0.14Å (Figure 2b), taking into account the estimated maximum coordinate error calculated by SFCHECK [21], which is sufficient for penetration of dioxygen.

As the substitutions introduced to the SgfSL did not change the fold of alpha-carbon chain in the active site, we reasoned that possible differences in activity must be attributed solely to the mutations themselves.

The structures of SgfSL, and its mutant forms with sufficient occupancy of copper ions, were obtained via the cultivation of the corresponding over-producer, the *E. coli* strain, in a medium with a high concentration of copper(II) sulfate (1 mM); in the case of cultivation with 0.25 mM CuSO_4_, the laccase crystal structures were deprived of copper (Table 2) [10].

Table 1, Table 2 and Table 3 represent the data related to the SgfSL mutants that were expressed in a medium with 1 mM CuSO_4_; SgfSL_low Cu_ and His165Phe_low Cu_ are the SgfSL and His165Phe variant produced in a medium supplemented with 0.25 mM CuSO_4_. In the case of low concentration of CuSO_4_, the structure of the mutant protein lacks not only copper Cu2, but also Cu3_β_.

We used ABTS and K_4_[Fe(CN)_6_] as electron donors and phenolic compound 2,6-DMP as combined electron and proton donor. The laccase oxidized ABTS and K_4_[Fe(CN)_6_] at pH 4.0, 2,6-DMP was oxidized at pH 9.0. Substrate affinity (K_m_), turnover rates (k_cat_) and catalytic efficiency (k_cat_/K_m_) values for each enzyme are listed in Table 3 and Figure S2. The specific activity of SgfSL toward phenolic compound was lower than toward electron donors, ABTS and K_4_[Fe(CN)_6_].

As a result, the substitutions of Ile170, and the mutation His165Phe (His165Phe_low Cu_ as well), dramatically affected the activity of the SgfSL. These mutants showed significantly reduced turnover number for ABTS, K_4_[Fe(CN)_6_]; and these variants were incapable of oxidizing 2,6-DMP. The catalytic efficiency of His165Ala variant was slightly higher than that of the SgfSL wt (Table 3).

Laccases are strongly inhibited by azide ions. The activity of 3D laccases was completely abolished by low concentrations of sodium azide (ranging from several μM to 1–2 mM) [22,23]. High resistance to inhibition by sodium azide was observed in 2D laccases previously [5]. We examined the effect of sodium azide on the activity of wild type SgfSL and its mutants. The inhibition assays were carried out at pH 4.0, using ABTS as a substrate. In the presence of 10 mM NaN_3_ activity of wild type SgfSL was decreased to 53.9%, whereas the activity of the His165Ala variant was increased by thirty percent.

The thermostability of His165Ala variant is as high as that of wild type SgfSL; it retained approximately 80% of its initial activity after the 40 min incubation at 80 °C.

## 3. Discussion

### 3.1. Ile170 Could Be Indirectly Involved in The Coordination of Cu3_α_ Ion within the TNC

We extensively studied the routes leading to the TNC of 2D laccase SgfSl. The inability to identify tunnels using the noble gases forced us to use point mutations in SgfSL. We assumed that there are two routes leading to TNC of 2DLac, which were potentially suitable for the passage of dioxygen or water and probably copper ions.

One of the proposed T3 tunnels is blocked by the Ile170. Replacing Ile170 to alanine and phenylalanine result in a strong decrease in catalytic activity by an order of magnitude (Table 3). The expansion of the proposed T3_α_ tunnel upon replacing isoleucine to alanine (Figure 2a) did not increase enzymatic activity. Apparently, the role of Ile170 is not related to modulating dioxygen access to the TNC through this tunnel. We assumed that Ile170 is rather indirectly involved in the coordination of Cu3_α_ ion within the TNC. Indeed, the detailed comparative analysis of the structures of the wild type SgfSL and its two mutant forms showed that Ile170 can affect (via hydrophobic interactions) the position and mobility of the His157, belonging to the 1st coordination sphere of the Cu3α.

It was observed that the Cu3_α_-Cu3_β_ distance in the TNC is highly variable [24], which is important for stabilizing coordination geometries and mediating proton transfer reactions [25,26] The change in the distance is observed when going from the oxidized to partially and fully reduced structures. In the reduced structure these distances are the shortest and decreased by approximately 1 Å [27].

In our laccase structures, the Cu3_α_-Cu3_β_ distance in the TNC varied by more than 1 Å, from 3.96 ± 0.10 ± 0.11 Å in the Ile170Phe to 5.24 ± 0.04 ± 0.08 Å in the wild type (the average value for monomers in an asymmetric unit with standard deviation and estimated maximum coordinate error). Therefore, Ile170 indirectly affects the location of theCu3_α_. In case of Ile170Ala substitution, Ala is unable to establish nonpolar interactions through its side chain and hence it does not interact with His157, which may cause excessive mobility of imidazole ring of His157. In case of the Ile170Phe replacement, the imidazole ring of His157 can be more strongly fixed (Figure 2c) than it is required for the enzyme functioning. Both replacements of Ile170, inhibit the reduction of oxygen to water, within the TNC and catalytic activity of SgfSL is decreased.

Thus, the structure-function relationship was established for the second coordination sphere residue that explains the decreased activity of SgfSL with substitutions of the Ile170.

### 3.2. Histidine 165 Is A “Gateway” of T3 Tunnel Leading to Cu3_β_ of T2/T3 Center

The shortest route from the protein surface to the TNC exists in a direction through a structurally conserved His165. This residue is located at the entrance of the proposed dioxygen tunnel. In the majority of 2D laccases structures access through this tunnel was blocked by His165. The same feature was observed in the 3D laccase from *Thermus thermophilus* (PDB entry 2XU9), opening and closing of the T3 tunnel can be provided by the mobility of the side chains of His137 (structural analog of His165) and Glu451. We considered that the replacement of the His165 (“gateway” of the proposed tunnel) to alanine could fix the tunnel in an open state, and the hydrophobic phenylalanine should close the tunnel (Figure 1).

In line with our hypothesis, His165Phe variant showed drastically reduced turnover rates for ABTS; its catalytic efficiency decreased by a factor of 255 compared to SgfSL wt. Oxygen consumption decreased 6-fold for ABTS and by one order of magnitude for K_4_[Fe(CN)_6_]; no activity was detected towards 2,6-DMP (Table 3).

His165Phe_low Cu_ was obtained from an overproducer strain cultivated with 0.25 mM CuSO_4_. Interestingly, not only Cu2 but also Cu3_β_ were absent from the structure of this mutant (PDB entry 5MKM). Recently, we have shown that the T3_β_ of SgfSL is accessible for copper ions during dialysis of the protein preparation [10]. We propose that shuttering the T3_β_ tunnel in the His165Phe mutant may impede copper ion access to the TNC. This would explain why saturation of TNC with copper ions could only be reached at the increased (1 mM) copper content in the growth medium (Table 2). The shuttering of the T3_β_ tunnel likely impedes the access of dioxygen to the TNC, which would be in agreement with the fact that both His165Phe and His165Phe_low Cu_ variants were inactive (Table 3). Thus the Phe at the position 165 may hinder the access of both dioxygen and Cu ions to the TNC via the T3_β_ tunnel (Figure 2c).

The His165Ala variant possessed a catalytic efficiency slightly higher than that of the wild type SgfSL: Oxygen consumption increased 1.7-fold for ABTS and 2.8-fold for K_4_[Fe(CN)_6_] (Table 3). This could be explained by the increased oxygen access to the TNC upon the change of His165 to Ala. We assumed that His165 can regulate the oxygen supply via short T3_β_ tunnel to TNC.

**Figure 3:**
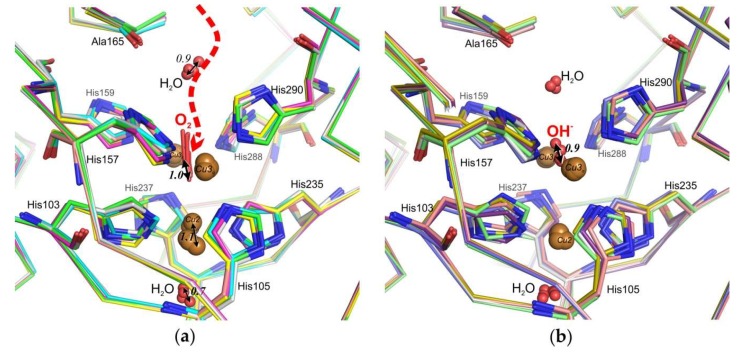
View of TNC at superposition of the monomers His165Ala of asymmetric unit. (**a**) “O_2_ –state”, (**b**) “Resting state”. The dotted line shows the T3_β_ tunnel. The distances between the extreme positions of atoms are shown in angstroms.

It is known that the different O_2_-reduction states and intermediates of 3D laccases could be observed in the crystals with different X-ray-absorbed doses [14,26]. In the case of the structure of the His165Ala variant, we noted movement of dioxygen and water molecules within the TNC in molecules of the asymmetric unit (Figure 3). We observed the “O_2_ –state” with symmetrically coordinated O_2_ amidst the binuclear T3 copper ions in 6 molecules of laccase (Figure 3a), oxidized “resting state” with bridging hydroxid (OH^−^) in 4 molecules (Figure 3b) and fully Cu reduced state without ligands between T3 copper ions in 2 molecules (not shown).

Superposition of 12 monomers in the asymmetric unit, using Cα atoms gave r.m.s.d. of 0.31± 0.14 Å, we obtained the same values for copper ions at the T3 center, indicating a good result at 2.3 Å resolution. However, positions of Cu2, water molecules, dioxygen, and hydroxyl ion varied within approximately 1Å, which allowed different stages of dioxygen penetration into the TNC and the hydroxyl release to be observed. (Figure 3).

It was shown recently that NaN_3_ reduced laccase activity of 2D laccases at acidic conditions, and no inhibition was observed at basic pH in the presence of up to 100 mM inhibitor [8]. It was observed that, in contrast to the wild type enzyme, the His165Ala variant demonstrates an increased activity under acidic conditions in the presence of 10 mM NaN_3_. The replacement of a positively charged His (at acidic pH) to non-polar Ala at the entrance to the single T3 tunnel reduces attraction of of the TNC for the negatively charged azide ions. We presently have no reliable explanation for activation of the His165Ala variant with azide at acidic pH. However, we would propose that azide ion can bind to 2D laccases at an alternative site, which could activate the enzyme to some extent. Indeed, we have recently demonstrated that in the structure of another 2D laccase *Streptomyces lividans* Ac 1709 (PDB ID 4GYB) azide is bound near the T1 center in the vicinity of the substrate-binding pocket. However, the mechanism of this phenomenon requires further research.

In summary, we suggest that the T3_β_ tunnel of 2D laccases can provide access of dioxygen, copper ions and inhibitor ions to the TNC. The investigations of accessibility of the TNC of 2D laccases can provide insights into the fundamental process of O_2_ reduction to H_2_O, and also will be important to the development of industrial and biomedical applications. Boosted activity and resistance to inhibitors of the His165Ala variant along with its high thermostability can be useful for industrial application of 2DLac.

## 4. Materials and Methods

### 4.1. Plasmid Construction

The pQE-30-based plasmid pQE-993nS carrying the gene encoding SgfSL deprived of the N-terminal signal sequence [10] was used as a template for the PCR with a pair of corresponding mutagenic primers (replaced nucleotides are in italic and underlined). Herein and after, the N-terminal truncated SgfSL will be referred to as the wild type (SgfSL wt) as the truncation does not affect the catalytic activity.

His165Ala_For: 5′- TCGGCACGGAA*GCG*GGCACGGGCGGCAT

His165Ala_Rev: 5′- ATGCCGCCCGTGCC*CGC*TTCCGTGCCGA

His165Phe_For: 5′- TCGGCACCGAA*TTT*GGCACCGGCGGCATTCGCAACGG

His165Phe_Rev: 5′- GCCGCCCGTGCC*AAA*TTCCGTGCCGACGACGTGGTC

Ile170Ala_For: 5′- GAACACGGCACCGGAGGA*GCG*CGCAATGGCCTGTATGGA

Ile170Ala_Rev: 5′- CGGTCCATACAGGCCATTGCG*CGC*TCCTCCGGTGCCGTGTTC

Ile170Phe_For: 5′-CGGCACCGGGGGG*TTT*CGCAACGGCCTGTACGG

Ile170Phe_Rev: 5′- CCGTACAGGCCGTTGCG*AAA*CCCCCCGGTGCCG

Amplification of target template was carried out by KOD Hot Start DNA Polymerase (Novagen, Germany), according to the manufacturer protocol. Then, the PCR products were digested with DpnI and used for the transformation of *Escherichia coli* DH5α competent cells.

As we failed to express SgfSL mutants I170A and I170F, using the obtained pQE-30-based vectors, these mutants were cloned in another expression vector, pET32-Xa_Lic. The genes of I170A and I170F SgfSL mutants were PCR-amplified using the corresponding pQE-30-based plasmids as a template, and the following pair of primers:

For 5′- AGATCTG*GGTACC*ATCGAGGGTCGTGCAGGAGCAGCGCCCGCCGGGGGA -3′

Rev 5′- GGCTGC*AAGCTT*TCAGTGAGCGTGCTCCTGCGGGT -3′

The PCR products obtained were digested with *Hind* III and Kpn I (Sibenzyme, Russia) and inserted to the pET32-Xa_Lic vector, treated with the same enzymes.

### 4.2. Purification of SgfSL Mutants

The *E. coli* strain M15 (pREP4) (Qiagen, Stockach, Germany) was transformed with the pQE-993nS, pQE30/H165A SgfSL or pQE30/H165F SgfSL. The *E. coli* strain BL21(DE3)/Rosetta (Qiagen, Stockach, Germany) was transformed with pET32/I170A SgfSL or pET32/I170F SgfSL. Cells were grown at 37 °C with shaking at 150 rev/min to OD_600_ = 0.5. The production of SgfSL variants was induced by addition of isopropyl β-D-1-thiogalactopyranoside (IPTG) to a final concentration of 0.1 mM. Along with IPTG, CuSO_4_ was added to a final concentration of 0.25 (low copper conditions) or 1 mM (high copper conditions). After induction, the cells were incubated for 18 h with shaking (50 rev/min) at either 18 °C (in case of H165A and H165F mutants) or 30 °C (in case of I170A SgfSL and I170F mutants).

In each case, cells were collected by centrifugation at 5000*g* for 15 min, suspended in buffer A (20 mM phosphate buffer pH 7.4, containing 0.5 M NaCl and 20 mM imidazole) with 1 mM phenylmethylsulfonyl fluoride and 200 ng/mL DNAase I and disrupted using EmulsiFlex-C3 high pressure homogenizer (Avestin, Ottawa, ON, Canada). Cell debris was removed by centrifugation (30 min at 15,000*g*) and the supernatant was loaded onto a column packed with Ni-NTA Agarose (Qiagen, Stockach, Germany) equilibrated with the buffer A. Then, the column was washed with four column volumes of the buffer A and the protein was eluted with the buffer A containing 150 mM imidazole.

For I170A and I170F mutants, fractions after Ni-NTA Agarose column were collected and dialyzed against a buffer for proteolysis (50 mM Tris-HCl, pH 8.0, 100 mM NaCl). Proteolysis was carried out for 16 h at room temperature by adding 1U factor Xa protease (SIGMA-ALDRICH, Taufkirchen, Germany) per 1 mg of recombinant protein and CaCl_2_ to 1mM. Following Ni-affinity chromatography made mutant proteins without thioredoxin N-terminal tail. For crystallization, all preparations of SgfSL variants were concentrated to 20–30 mg/mL and dialyzed against 50 mM H_3_BO_3_-NaOH, pH 9.0, 100 mM NaCl.

### 4.3. Activity Assays

The optimum pH for laccase activity was determined at 30 °C using a 50 mM Britton–Robinson buffer within the pH range 2.5–5.5 for 2,2-azino-bis-(3-ethylbenzthiazoline-6-sulfonate)-ABTS and within the pH range 6–10 for 2,6-dimethoxyphenol–2,6-DMP. The reaction mixture contained 1 mM substrate (ABTS or 2,6-DMP) and the enzyme variant (0.7–5 μg/mL). The laccase activity was determined as the amount of ABTS oxidation at 30 °C for 30 sec at the optimum pH.

Oxygen consumption was monitored at 30 °C in 50 mM Britton–Robinson buffers with pH optimum (4.0) with a Clark-type oxygen electrode (Hansatech, Norfolk, UK) for 60 sec; the concentrations of the substrate (ABTS or K_4_[Fe(CN)_6_]) and the enzyme variants were 1 mM, and 10 µg/mL, respectively.

Molar extinction coefficients were used to determine the reaction products were ε_469_ = 49,600 M^−1^ × cm^−1^ for 2,6-DMP and ε_420_ = 36,000 M^−1^ × cm^−1^ for ABTS [28,29]. It was taken into account that the enzyme is a homotrimer. All kinetic experiments were performed in triplicate. The resulting curves were fitted to the Michaelis–Menten equation by non-linear least-square regression with Origin 9.1 (Figure S2). The rate of oxygen consumption was determined using OxyGraph software systems (Hansatech, Norfolk, UK).

To test thermostability, a laccase variant (1 mg/mL in the Britton–Robinson buffer at optimal pH) was incubated at 80 °C for 40 min. After incubation, samples were immediately chilled on ice. Residual activities were calculated taking the initial activity of the enzyme as 100%.

To study the inhibition of laccase variants by sodium azide, the measurements were performed at 30 °C in 50 mM Britton–Robinson buffer with optimal pH, 10 mM NaN_3_ and enzymes at a concentration of 1 µg/mL. The residual activity was calculated taking the initial activity of the enzyme without NaN_3_ as 100%.

### 4.4. Crystallization and Crystallography

Crystallization experiments were performed at 23 °C using the hanging-drop vapour-diffusion method on siliconized glass cover slides in Linbro plates (Molecular Dimensions, Suffolk, UK). Drops were made by mixing 2 µL of protein at a concentration of 10–20 mg/mL in 0.1 M NaCl, 0.05M H_3_BO_3_-NaOH pH 9.0 and 1 µL of reservoir solution.

The reservoir solution consisted of 20% PEG 6000, 0.1 M Bicine, pH 9.0 for H165A and H165F variants and 25% Smear medium PEG, 0.1 M Bicine, pH 9.0 (condition #21 of BCS-2 from Molecular Dimensions, Suffolk, UK) for I170A and I170F variants. For diffraction data collection, a single crystal was flash cooled after soaking in a solution consisting of 25% PEG 4000, 15% glycerol, 0.1 M Tris–HCl pH 8.0 prior to data collection.

Diffraction data for wild type, H165F, H165A, I170F and I170A variants have been collected on the beamline BL14.1 at the BESSY II electron storage ring operated by the Helmholtz-Zentrum Berlin [30] using Pilatus 6M detector [31]. Data for H165F_low Cu_ were collected at the beamline P11 (PETRA III, Hamburg). All data were processed and merged using the *XDS* package [32]. Crystallographic data statistics are summarized in Table 1.

The structures were determined by molecular replacement with Phaser [33] using the structure of a mutant laccase from *S. griseoflavus* determined at 1.9 Å resolution (PDB entry 5O4Q [10]) as a search model. Water molecules and metal ions were removed from the model. The initial model was subjected to crystallographic refinement with *REFMAC5* [34]. Manual rebuilding of the model was carried out in *Coot* [35]. The final refinement cycle with refinement of occupancy with the copper ions was performed in *Phenix* [36]. Data and refinement statistics are summarized in Table 1. The atom coordinates and structure factors have been deposited in the Protein Data Bank. Figures were prepared using *PyMOL* [37]. The tunnels leading from the protein surface to the TNC were calculated using *Caver* [20].

### 4.5. Noble Gas Pressurization

Prior to data collection, the crystals were incubated with xenon at 10, 15, and 30 bar, and with krypton at 40 bar, for 2–20 min in a Xenon Chamber (Hampton Research, Aliso Viejo, CA, USA) and after that flash frozen in liquid nitrogen at BESSY. We also used the technique “soak-and-freeze” developed in the ESRF by Carpentier [19], and worked on crystals by krypton at pressures from 142 to 150 Bar.

## Figures and Tables

**Table 1:** Crystallographic Data Collection and Refinement Statistics.

	H165F_low Cu_	H165F	H165A	I170F	I170A	SgfSLwt_low Cu_
**Data collection**						
Space group	P 2_1_	P1	P1	P1	P 2_1_	P 2_1_
Cell parameters						
a, b, c (Å)	75.88, 95.07, 116.82	77.03, 94.58, 115.93	77.36, 95.12, 116.48	77.25, 94.77, 116.18	74.79, 94.69, 116.49	75.53, 94.62, 116.50
α, β, γ (°)	90.00, 90.58, 90.00	90.00, 90.00, 92.01	90.13, 90.11, 91.85	90.13, 89.95, 92.56	90.00, 90.87, 90.00	90.00, 90.39, 90.00
Monomers in asymmetric unit	6	12	12	12	6	6
Resolution (Å)	50.0–2.0 (2.12–2.0) ^a^	50.0–1.92 (2.05–1.92) ^a^	50.0–2.3 (2.45–2.30) ^a^	50.0–1.85 (1.90–1.85) ^a^	50.0–1.98 (2.03–1.98) ^a^	50.0–1.80 (1.84–1.80) ^a^
Total No. of reflections	206,927 (29,456)	833,269 (147,319)	507,815 (80,770)	536,185 (26,532)	757,980 (54,090)	574,463 (45,966)
No. of unique reflections	90,309 (14,288)	235,591 (80,443)	143,173 (22,664)	256,815 (12,984)	112,783 (8272)	151,965 (11,814)
Rmerge (%)	10.5 (50.8)	9.1 (86.0)	11.0 (83.8)	8.2 (88.7)	12.5 (87.5)	6.6 (88.2)
I/σ (I)	5.41 (1.49)	7.88 (1.57)	9.35 (1.42)	5.89 (0.96)	10.13 (2.30)	9.86 (1.55)
Completeness (%)	80.2 (78.9)	94.3 (92.9)	98.0 (95.9)	91.4 (62.2)	99.8 (99.9)	99.4 (99.7)
CC_1/2_	0.99 (0.69)	0.99 (0.63)	0.99 (0.57)	0.99 (0.35)	0.99 (0.73)	0.99 (0.67)
Redundancy	2.29 (2.06)	3.59 (3.55)	3.55 (3.57)	2.09 (2.04)	6.72 (6.54)	3.78 (3.89)
**Refinement**						
Resolution (Å)	49.77–2.00 (2.02–2.00)	49.41–1.95 (1.98–1.95)	49.7–2.3 (2.34–2.31)	47.33–1.85 (1.87–1.85)	49.60–1.98 (2.01–1.98)	45.97–1.80 (1.84–1.80)
No. reflections	90,303 (5785)	225,948 (20,608)	143,173 (6693)	256,747 (5256)	112,768 (3514)	150,800 (10,599)
Rwork (%)	19.97 (31.94)	19.47 (30.86)	17.09 (26.66)	17.85 (35.78)	17.40 (24.36)	14.20 (27.78)
R_free_ (%)	23.32 (35.91)	23.10 (32.81)	22.21 (30.83)	23.70 (39.29)	21.48 (29.57)	17.94 (32.88)
R.m.s. deviations						
Bond lengths (Å)	0.008	0.008	0.008	0.008	0.007	0.007
Bond angles (°)	0.928	0.995	0.961	0.874	0.854	0.823
Ramachandran plot (%)						
Most favored	96.91	96.78	94.66	96.40	98.07	97.82
Additionally allowed	3.09	3.07	4.88	3.60	1.93	2.18
Generously allowed	0.00	0.15	0.46	0.00	0.00	0.00
PDB ID	5MKM	6FC7	6FDJ	6RH9	6RHQ	6S0O

Data were collected at 100K. ^a^ Values in parentheses are for highest-resolution shell.

**Table 2:** Occupancy and B-factor of copper ions.

Protein	Cu1		Cu3_α_		Cu3_β_		Cu2	
	Occupancy * (%)	B-factor * (Å^2^)	Occupancy * (%)	B-factor * (Å^2^)	Occupancy* (%)	B-factor * (Å^2^)	Occupancy * (%)	B-factor * (Å^2^)
SgfSLwt	0.98	19	1.00	21	1.00	24	0.48	56
SgfSLwt_low Cu_	1.00	31	1.00	39	0.88	48	0.22	73
H165F_low Cu_	1.00	22	0.92	28	0	0	0	0
H165F	0.96	27	0.96	34	0.70	38	0.27	40
H165A	0.98	28	0.99	35	0.95	40	0.48	40
I170F	1	31	1	35	0.97	39	0.53	100
I170A	1	25	0.97	31	0.75	38	0.19	50

*—the average number of copper ions in the asymmetric unit.

**Table 3:** Kinetic parameters of the SgfSL variants.

Substrate	Protein	K_m_ (mM)	k_cat_ (s^−1^)	k_cat_/K_m_ (mM^−1^ s^−1^)	Activity nM O_2_ min^−1^ µg^−1^
ABTS	SgfSL WT	0.10 ± 0.008	6.60 ± 0.12	66.28	0.82 ± 0.01
	H165A	0.12 ± 0.01	10.80 ± 0.21	90.00	1.45 ± 0.10
	H165F	0.45 ± 0.03	0.12 ± 0.009	0.26	0.13 ± 0.06
	H165F_low Cu_	0.47± 0.02	0.11 ± 0.007	0.23	0.10 ± 0.03
	I170A	0.63 ± 0.11	0.70 ± 0.20	1.11	0.11 ± 0.01
	I170F	0.20 ± 0.04	0.08 ± 0.01	0.40	0.08 ± 0.04
2,6-DMP	SgfSL WT	5.20 ± 0.11	0.80 ± 0.09	0.15	
	H165A	6.10 ± 0.34	1.10 ± 0.06	0.18	
	H165F	-	-	-	
	H165F_low Cu_	-	-	-	
	I170A	-	-	-	
	I170F	-	-	-	
K_4_ [Fe(CN)_6_]	SgfSL WT				2.28 ± 0.17
	H165A				6.47 ± 0.27
	H165F				0.26 ± 0.12
	I170A				0.18 ± 0.01
	I170F				0.14 ± 0.04

- no activity.

## Supplementary Materials

Supplementary Materials can be found at https://www.mdpi.com/1422-0067/20/13/3184/s1.

## Abbreviations

TNC	TriNuclear Center
3D	Three-domain
2D	Two-domain
SgfSL	*Streptomyces griseoflavus* Small Laccase
