# Supplementary material for: Investigations of Accessibility of T2/T3 Copper Center of Two-Domain Laccase from Streptomyces griseoflavus Ac-993
Source: Int J Mol Sci. 2019 Jun 28;20(13):3184. doi: 10.3390/ijms20133184 (PMC6650940; doi:10.3390/ijms20133184)
Supplement: Supplementary file 1 [file ijms-20-03184-s001.pdf]

**Supplementary Material**

Table S1. Ligands within the TNC of SgfSLwt and its mutant forms.

| **Protein** | Monomers in asymmetric unit | Number of Ligands | | | |
|---|---|---|---|---|---|
| | | OXY | PER | $OH^-$ / $H_2O$ | No ligand |
| SgfSLwt | 6 | 6 | - | - | - |
| $SgfSLwt_{low\ Cu}$ | 6 | - | 2 | 4 | - |
| $H165F_{low\ Cu}$ | 6 | 2 | - | 4 | - |
| H165F | 12 | 2 | 6 | 4 | - |
| H165A | 12 | 6 | - | 4 | 2 |
| I170F | 12 | 1 | - | 11 | - |
| I170A | 6 | - | - | 6 | - |

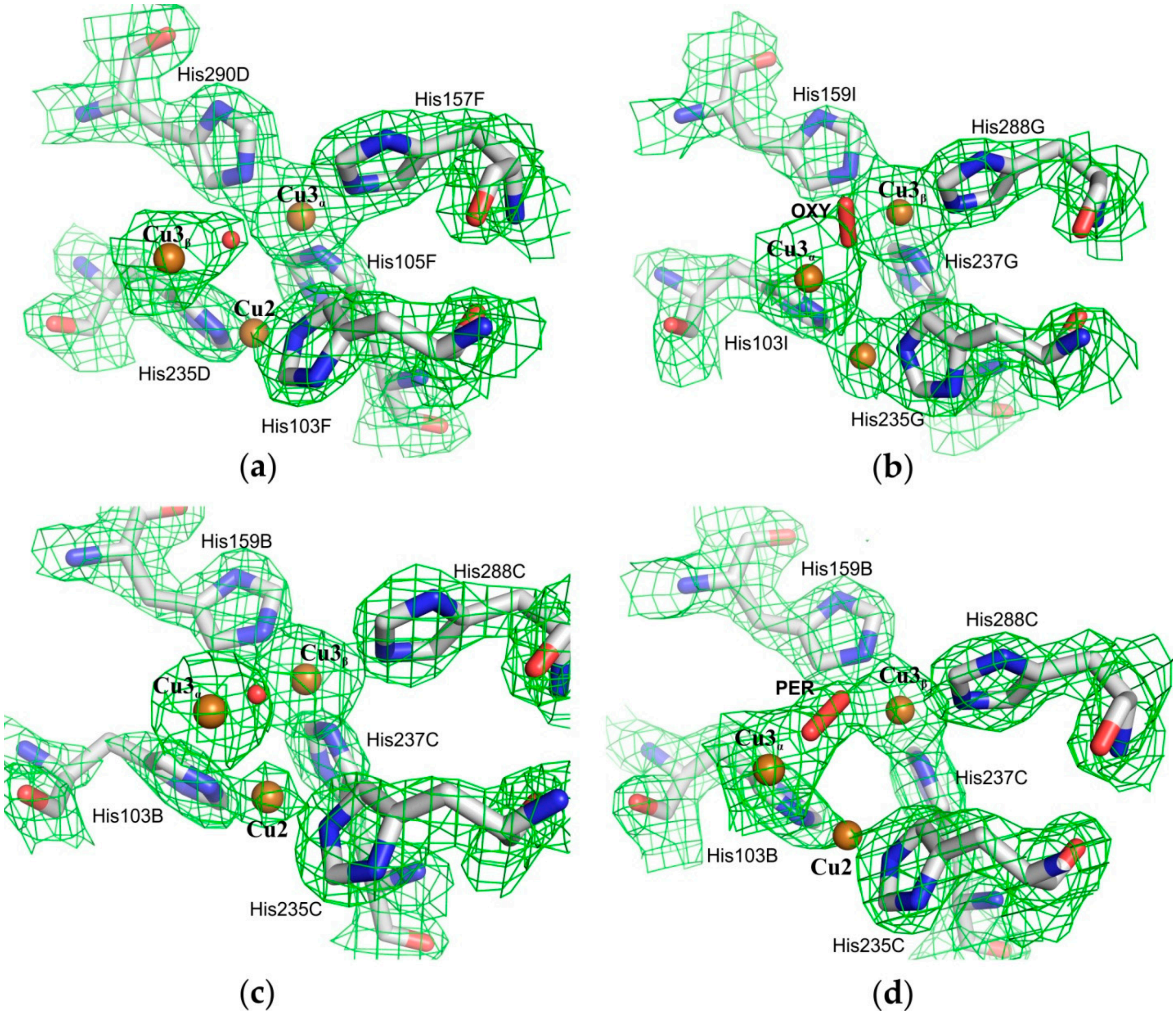


**Figure S1**. Fragments of $2F_o$-$F_c$ electron density maps around of the T2/T3 center of SgfSL Ile170Ala variant at 1.5$\sigma$ (**a**), His165Ala at 1.6$\sigma$ (**b**), Ile170Phe at 2.0$\sigma$ (**c**) and His165Phe at 1.5$\sigma$ (**d**). PER - peroxide ion, OXY - dioxigen. Copper ions are shown as spheres.

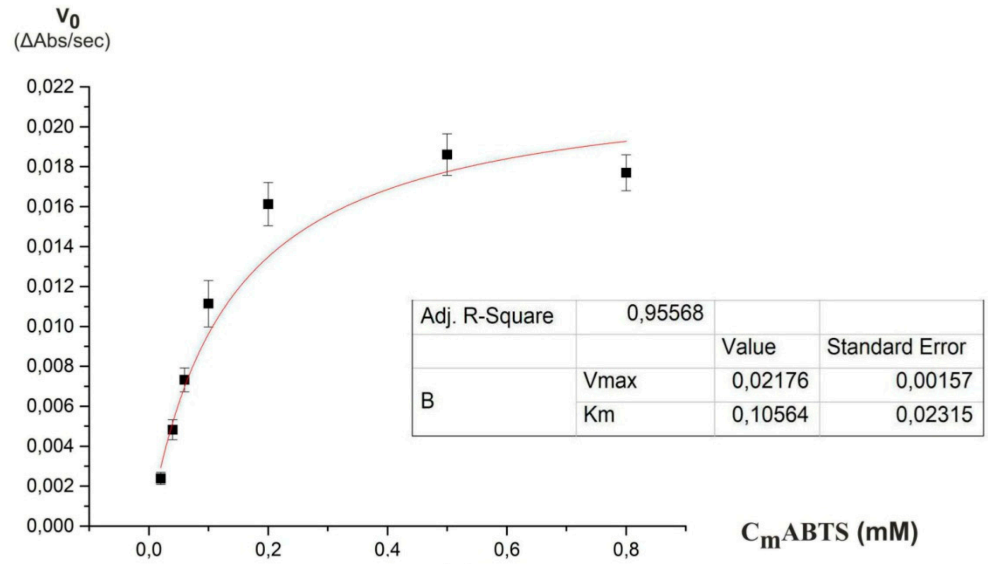
v0
(ΔAbs/sec)
0,022
0,020
0,018
0,016
0,014
0,012
0,010
0,008
0,006
0,004
0,002
0,000
0,0
0,2
0,4
0,6
0,8
CmABTS (mM)
Adj. R-Square 0,95568
Value
Standard Error
B
Vmax 0,02176 0,00157
Km 0,10564 0,02315

(a)

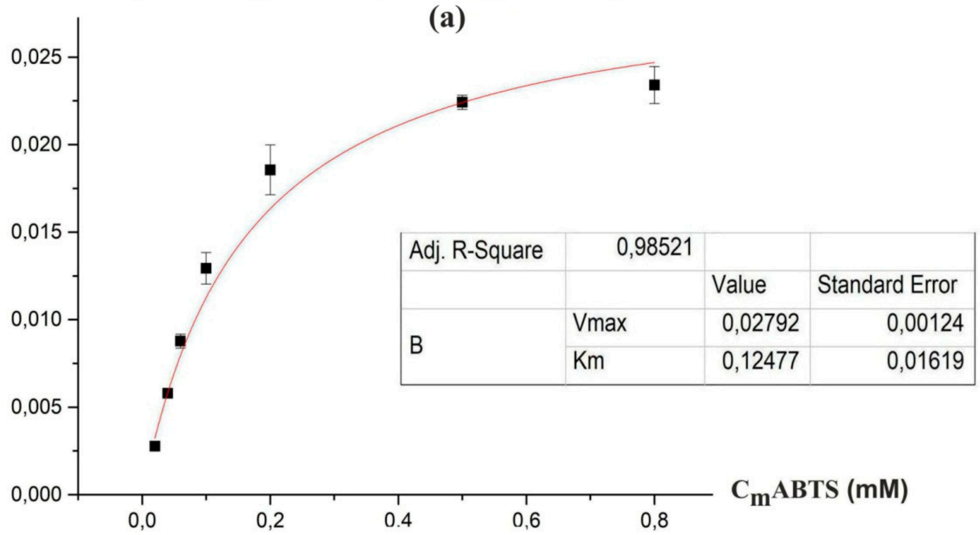
0,025
0,020
0,015
0,010
0,005
0,000
0,0
0,2
0,4
0,6
0,8
CmABTS (mM)
Adj. R-Square 0,98521
Value
Standard Error
B
Vmax 0,02792 0,00124
Km 0,12477 0,01619

(b)

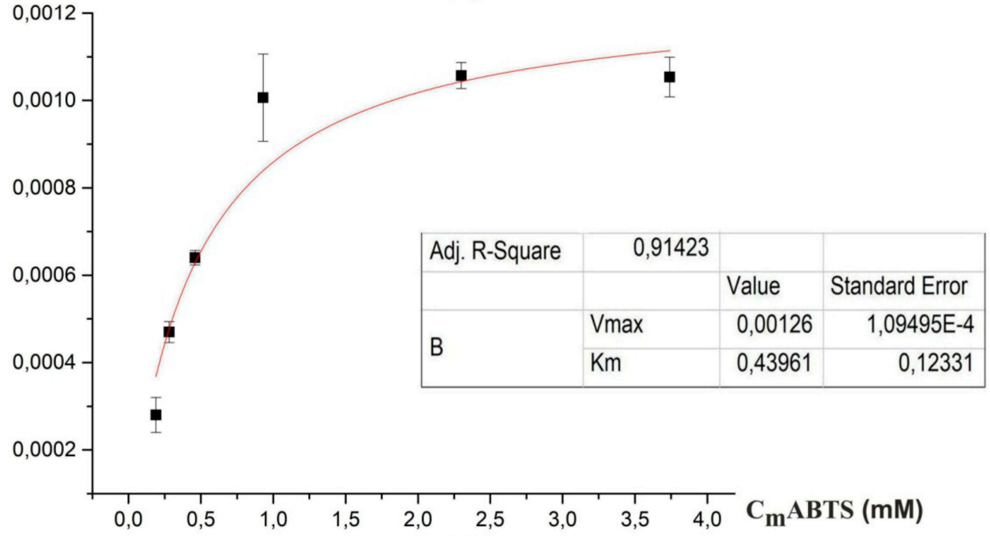
0,0012
0,0010
0,0008
0,0006
0,0004
0,0002
0,0
0,5
1,0
1,5
2,0
2,5
3,0
3,5
4,0
CmABTS (mM)
Adj. R-Square 0,91423
Value
Standard Error
B
Vmax 0,00126 1,09495E-4
Km 0,43961 0,12331

(c)

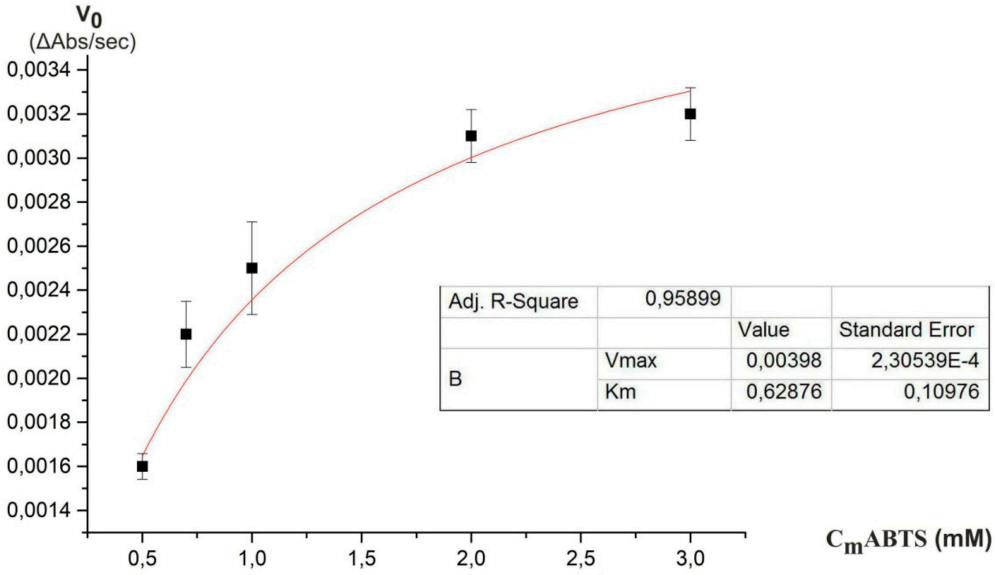


**(d)**

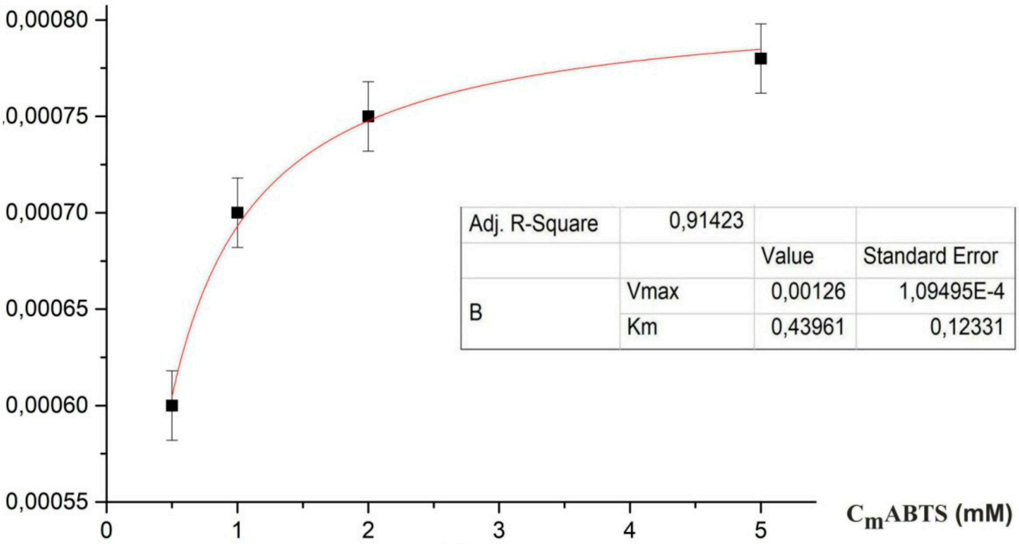


**(e)**

**Figure S2**. The graphs for determining kinetic parameters for different variants of SgfSL enzyme using ABTS as substrate. SgfSL wt(**a**), His165Ala (**b**), His165Phe (**c**) Ile170Ala (**d**) and le170Phe (**e**). Dots on the graphs represent the average values of three independent measurements. Curves are calculated using the Origin program.
